# Successful treatment of a painful os peroneum using conservative measures, infiltration therapy, and shock waves

**DOI:** 10.1093/jscr/rjad645

**Published:** 2023-12-06

**Authors:** Julian Ramin Andresen, Stephan Puchner, Sebastian Radmer

**Affiliations:** Department of Orthopedics and Trauma Surgery, Medical University of Vienna, Vienna, Austria; Department of Orthopedics and Trauma Surgery, Medical University of Vienna, Vienna, Austria; Specialist Practice for Orthopedics, Centre for Orthopaedics, Berlin, Germany

**Keywords:** accessory tarsal bones, lateral foot pain, os peroneum, painful os peroneum syndrome, infiltration therapy, shockwave therapy

## Abstract

Lateroplantar foot pain may be caused by various entities, whereby painful os peroneum syndrome should be included in the differential diagnosis. Physical examination and multimodal imaging enable a definitive diagnosis. We report on a 59-year-old man with severe, load-dependent pain, corresponding to an os peroneum syndrome, triggered by a pes planovalgus with consecutively induced focal inflammation and tendovaginitis of the tendon of the peroneus longus muscle. Multifactorial conservative measures including infiltration and shockwave therapy finally led to a restoration of the original condition.

## Introduction

The os peroneum is a roundish oval accessory bone, which is located as a sesamoid bone in the distal half of the tendon of the peroneus longus muscle on the plantar side of the os cuboideum [1]. Along with the os trigonum and the os tibiale externum, the os peroneum is one of the most common accessory bones in the tarsal region and is found in the normal foot skeleton with an incidence of 3.9%–30% [1–4], depending on the literature. In 30% of cases, the os peroneum appears in two parts and in 60%, it is bilateral [5]. The peroneal bone is usually asymptomatic, but both trauma and mechanical irritation of the surrounding soft tissues and tendons can cause lateroplantar foot pain, which is then referred to as painful os peroneum syndrome (POPS) [5–7]. In conditions following trauma, the os peroneum should not be confused with a bony avulsion, although a fracture of the os peroneum itself is also possible [5, 8].

We report on an athletic patient with subacute to chronic lateroplantar foot pain without previous trauma, which led to consecutive immobilization.

## Case report

**Figure 1:**
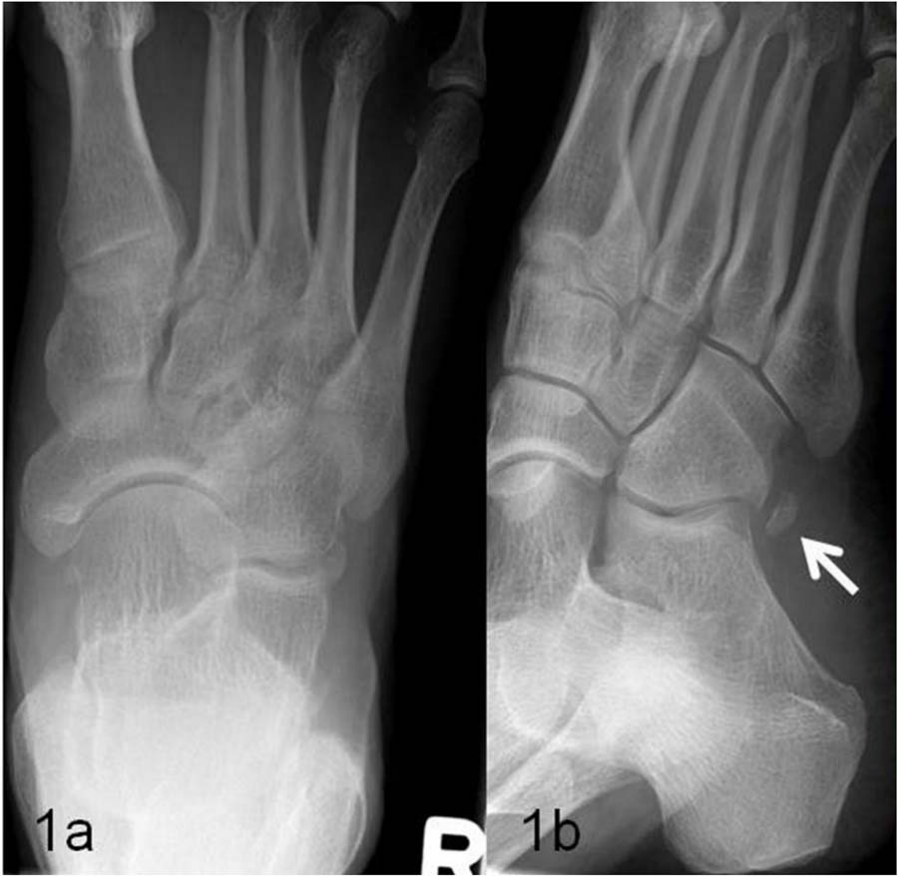
(a, b) Presentation of the os peroneum (marked with an arrow) in the oblique X-ray image.

**Figure 2:**
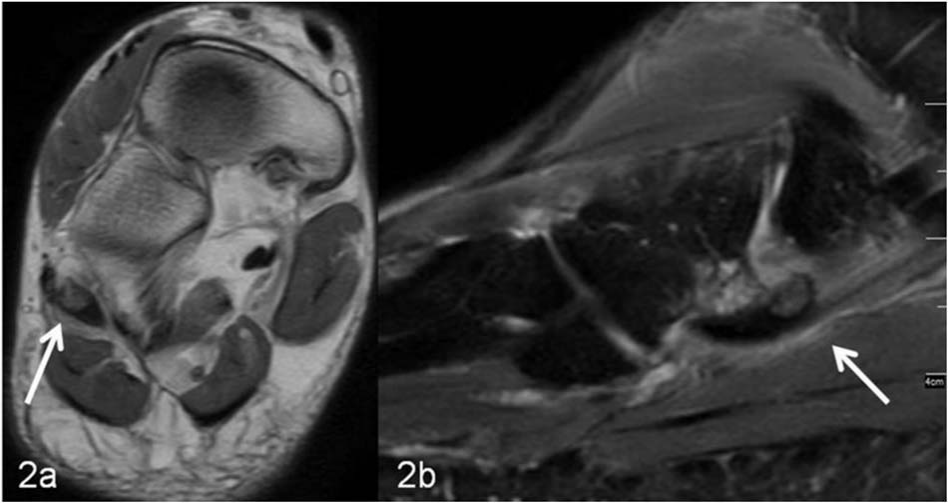
(a, b) MRI imaging, (a) axial T1 weighted slice, the os peroneum is centrally located in the long peroneal tendon (marked with an arrow). In (b) sagittal T2 weighted fat-suppressed slice, around the os peroneum (marked with an arrow) and along the tendon there is a marked signal enhancement, corresponding to inflammatory fluid accumulation (tenosynovitis).

A 59-year-old man reported severe, load-dependent pain (7 out of 10 score points on the VAS) of his right foot, increasing over a period of about 1 month. He had no memory of having experienced trauma. Clinically, there was a pes planovalgus, a slight swelling, and warm sensation distolaterally of the malleolus lateralis over the course of the tendon of the peroneus longus muscle, where there was also clear pain upon pressure, and forced plantar flexion was very painful. For imaging diagnostics, conventional X-rays were taken, which revealed an elongated oval os peroneum with a size of 9.5 × 5 mm in a typical location (Fig. 1a and b). Furthermore, an MRI examination was performed, which revealed a clear perifocal soft tissue oedema of the os peroneum with involvement of the peroneus longus muscle, whereas a tendon rupture was ruled out (Fig. 2a and b).

Therapeutically, the foot was relieved and immobilized with a lower leg foot orthosis (Vacoped-Orthese*®*) for 6 weeks. Supportive analgesic/antiphlogistic systemic therapy with a nonsteroidal anti-inflammatory drug (etoricoxib 90 mg, Etoricoxib-ratiopharm®) was given once a day for 14 days. The patient was also instructed to apply cryotherapy several times a day according to instructions.

As there was no relevant clinical improvement, an additional ultrasound-guided infiltration therapy (injection of 2 ml of a drug mixture consisting of 1 ml of 2.5 mg dexamethasone (Lipotalon*®*) and 1 ml bupivacaine (Carbostesin® 0.5%) with three repetitions at intervals of 7 days) was performed, which led to a slight reduction of the symptoms.

**Figure 3:**
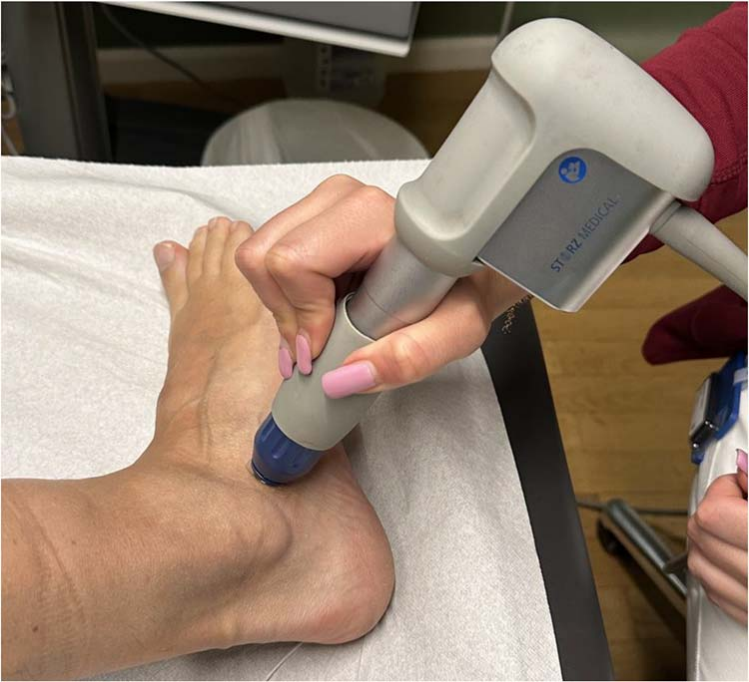
Illustration of the technical implementation of extracorporeal shockwave therapy.

Thereafter, an extracorporeal radial shockwave therapy of 4000 pulses, 1 bar, 20 Hz (STORZ MEDICAL, Duolith SD1) was performed five times at intervals of 3 days (Fig. 3), which resulted in a marked and sustained pain relief.

After the removal of the orthosis, physiotherapeutic treatment with manual therapy and physiotherapy was initiated, and insoles were fitted to compensate for the existing foot deformity.

After the completion of the therapeutic measures, 9 weeks after initial presentation, the patient was free of pain and could put weight on his foot without restriction, and he was able to participate in recreational sports again without any problems. No recurrence developed over a follow-up period of another 6 months.

## Discussion

Acute or chronic load-dependent pain in the lateral foot is not uncommon in adults, and the causes are varied. If there is painful restriction of movement with increasing pain on palpation above the os cuboideum and focal warming, POPS should also be considered [5, 7, 9].

In addition to conventional X-rays, ultrasound and MRI are used for differential diagnosis and treatment planning. A dislocation, fracture, hypertrophic, or multisegmental form of the peroneal bone, with or without accompanying perifocal irritation and tenosynovitis or rupture of the long peroneal tendon, can be visualized effectively [7–13]. In our patient, an os peroneum of normal size and localization was found on conventional X-ray (Fig. 1a and b). In the MRI examination, there was clear perifocal soft tissue oedema with a tenosynovitis of the tendon of the peroneus longus muscle (Fig. 2a and b). At the same time, the arch of the foot was lowered and there was a marked pes planovalgus, so that, when everything was taken into consideration, it was assumed that our patient had subacute/chronic postural stress with accompanying perifocal inflammation.

For this constellation, conservative measures with immobilization and analgesic/anti-inflammatory medication with nonsteroidal anti-inflammatory drugs are initially recommended [14], which can lead to freedom from symptoms even in the case of a fracture of the os peroneum [15].

At the same time, a customized shoe insole corrects the foot malposition, in our patient with pes planovalgus eliminating the root cause [9].

In order to further reduce the inflammation, an ultrasound-guided steroid injection, which is easy to perform in the lateral foot region, was given because of persistent symptoms [16]. It usually takes 1–2 weeks for the injection to take full effect, but this did not lead to any relevant breakthrough in our patient. Risks to consider with the injection are a rise in blood glucose levels in diabetics, a postinjection flash or infection as rare events. With good anatomical knowledge of the foot anatomy, the infiltration can also be performed safely under image converter control [17].

A subsequent shockwave therapy led to marked and sustained pain relief. With radial shock waves, low-energy waves spread over a wide area and stimulate the formation of cytokines in the cells, cell metabolism is increased, and the tissue in the treated region is supplied with more blood, which promotes the healing process. The application is usually slightly painful. Overall, shockwave therapy is an effective and safe noninvasive procedure in the treatment of tendopathies [18]. In the area of the foot, most experience has been gained in the treatment of chronic plantar fasciitis, with good results [19]. With the exception of one case [9], there are no further reports on its specific use to treat POPS.

Surgical tendon reconstruction was not indicated in our case, as no rupture or tearing of the long peroneal tendon was found on MRI imaging.

## Conclusion

In the case of lateroplantar foot pain and presentation of an os peroneum, a POPS should also be considered in the differential diagnosis. The cause may be a flattening of the arch of the foot, which can be corrected with adapted shoe insoles. An accompanying inflammatory reaction of the region of the os peroneum and the long peroneal tendon can be suppressed by immobilization, anti-inflammatory medication, steroid infiltration, and shockwave therapy, whereby in our case, complete and sustained freedom from symptoms was only achieved after shockwave therapy. In the therapeutic cascade for the treatment of POPS, the possibility of shockwave therapy should be considered in the case of a non-ruptured tendon of the peroneus longus muscle.
